# Single-contig bacterial genomes recovered from cattle fecal metagenomes at farms with variable antibiotic use

**DOI:** 10.21203/rs.3.rs-9715194/v1

**Published:** 2026-06-05

**Authors:** Priscila Thiago Dobbler, Anitha Ravi, Tomáš Větrovský, Eva Pěchoučková, Alexandr Nemec, Martina Kyselková

**Affiliations:** Institute of Microbiology of the Czech Academy of Sciences; Institute of Microbiology of the Czech Academy of Sciences; Institute of Microbiology of the Czech Academy of Sciences; Czech University of Life Sciences Prague; National Institute of Public Health; Institute of Microbiology of the Czech Academy of Sciences

## Abstract

Cattle feces represent a complex microbial reservoir with implications for animal health and the environmental dissemination of microorganisms and antibiotic resistance genes. Metagenomic studies have shown that cattle fecal communities are dominated by Bacillota and Bacteroidota, whereas low-abundance taxa, including potential pathogens, often remain underrepresented due to methodological detection limits. Here, we present 84 single-contig, medium- to high-quality metagenome-assembled genomes (MAGs) recovered from cattle feces after enrichment for bacteria able to grow in acetate-supplemented minimal medium. The MAGs were classified within the phyla Actinomycetota (20 MAGs), Bacillota (5), Bacteroidota (21), Patescibacteriota (5), and Pseudomonadota (33), with 41 MAGs representing putative novel taxa at species to family level. Nineteen MAGs carried antibiotic resistance genes and six MAGs were assigned to opportunistic pathogenic species. This dataset thus provides a genomic resource for studies of bacterial diversity and antimicrobial resistance at the animal–environment interface within a One Health framework.

## Background & Summary

The cattle gastrointestinal microbiota contribute to bovine digestion and immune function and thereby influence animal health and food quality^1^. The cattle fecal microbiome additionally affects human and environmental health through zoonotic transmission and the dissemination of antibiotic resistance genes following the application of cattle manure to agricultural fields^2^. Knowledge of the composition and function of the bovine gastrointestinal microbiome has increased substantially in recent years, largely through high-throughput sequencing approaches^3^, including metagenome-assembled genomes (MAGs), which directly link taxonomic identity with functional potential^4,5^. However, existing MAG collections from cattle gastrointestinal tract predominantly represent abundant bacterial taxa, mainly Bacteroidota and Bacillota, whereas low-abundance groups remain underrepresented^5^. Despite their low abundance, such bacteria may play important roles in the digestion of plant-derived material and may include opportunistic pathogens^6^.

Bacterial enrichment cultures represent an effective approach for recovering low-abundance members of complex microbial communities such as soil or manure^7,8^. In our associated study, cultures of fresh cattle feces were established using a mineral medium with acetate as the sole carbon source to enrich for diverse *Acinetobacter* species^8^. However, this medium is not fully specific for acinetobacters and may also support the growth of other bacteria, particularly when residual nutrients from the original, nutrient-rich material, such as cattle feces, are present. Shotgun metagenomic sequencing of the enrichment cultures thus enabled the recovery of both *Acinetobacter* MAGs, which were analyzed in our associated study^8^, and non-*Acinetobacter* MAGs, which are presented here.

In this Data Descriptor, we present 84 non-*Acinetobacter* MAGs that were recovered from cattle feces after enrichment in minimal medium supplemented with acetate. The reduced diversity of the enrichment cultures enabled the recovery of single-contig MAG assemblies, including highly complete MAGs, without the need for binning. This represents an advantage for confidently linking antibiotic resistance genes and other functional traits to their host genomes, which can be compromised in binned MAGs due to their frequent chimerism^9^.

Of the 84 MAGs, all but seven MAGs contained at least one copy of each ribosomal RNA gene class (5S, 16S, and 23S), and all encoded at least 14 distinct tRNA types. Twenty-two MAGs represented near-complete genomes, with ≥ 99% completeness, < 1% contamination, at least one copy of each ribosomal RNA class, and 23–26 tRNA types. An additional 11 MAGs satisfied the high-quality MAG standards proposed by Bowers et al.^10^, while the remaining 51 MAGs were classified as medium-quality MAGs, with a maximum contamination level of 2.34% (Supplementary Table 1).

**Figure 1:**
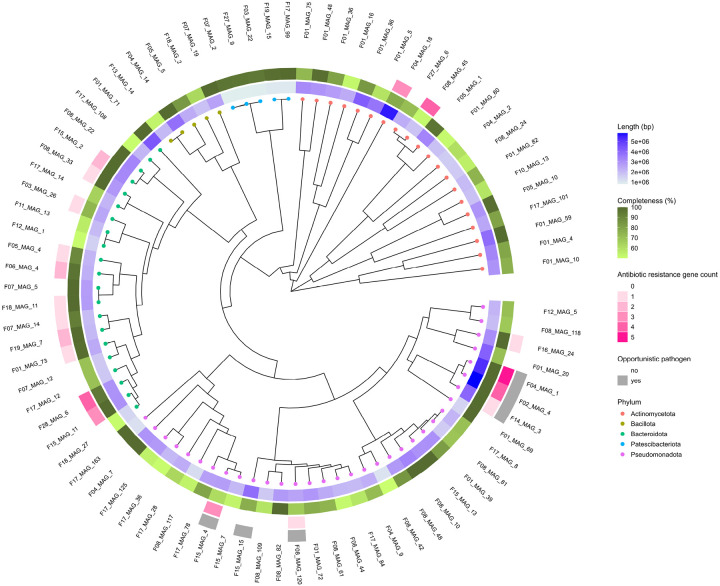
Genome-based phylogenetic tree showing the relationships among the 84 non-*Acinetobacter*single-contig MAGs recovered from acetate-enriched cattle fecal communities. Tip colors indicate GTDB phylum assignments. Concentric rings summarize assembly length, genome completeness, and the number of antibiotic resistance genes (from inner to outer rings; see color legend for details). Grey squares in the outermost ring indicate MAGs assigned to species reported from opportunistic human infections. MAG labels correspond to the MAG IDs listed in Supplementary Table 1.

Based on taxonomic classification provided by GTDB-Tk^20^ using GTDB release R10-RS226^24^, most MAGs were assigned to Pseudomonadota (33 MAGs), followed by Bacteroidota (21 MAGs), Actinomycetota (20 MAGs), Bacillota (5 MAGs), and Patescibacteriota (5 MAGs). In total, 35 MAGs represented putative novel species-level taxa, three corresponded to putative novel genus-level taxa, and three, all within Patescibacteriota, could not be assigned to any family and were classified only at the order level (Fig. 1, Supplementary Table 1). Among the species with validly published names represented in our dataset, *Alcaligenes faecalis, Bordetella trematum, Brucella intermedia, Escherichia coli*, *Klebsiella grimontii*, and *Pantoea agglomerans* are associated with infections in humans^11–16^. Nineteen MAGs harbored antibiotic resistance genes, with a maximum of five such genes per genome (Fig. 1, Supplementary Table 1).

Collectively, this resource will facilitate studies of microbial diversity, genome evolution, and host–microbiome interactions. It may inform risk assessment and surveillance strategies at the interface of animal and human health.

## Methods

### Ethics statement

The study involved only the collection of bovine fecal samples from the farm floor. Therefore, in accordance with Czech and EU regulations, no formal animal experiment approval was required for this study. Sampling was conducted with the consent of the farm owners.

### Sampling and performing bacterial enrichments

Cattle feces sampling and performing bacterial enrichments were described earlier in the associated study^8^. In brief, cattle feces were sampled at 28 anonymous Czech cattle farms representing varying levels of antibiotic use during 2022. At each farm, a composite sample consisting of 5–10 dung subsamples was collected from the farm floor or pasture using a sterile garden trowel while avoiding direct contact with the ground. After thorough mixing of the samples in their sampling bags, 2 g aliquots were mixed with 2 mL of 0.9% saline and 4 mL of glycerol and stored at − 20°C. To establish the enrichment cultures, each sample was washed and added to a 100-mL Erlenmeyer flask with 25 mL liquid mineral medium supplemented with 0.5% (w/v) sodium acetate (ACE^17^). The cultures were incubated at 30°C with shaking at 160 rpm for 3 h, followed by passive sedimentation for 30 min. Subsequently, 5 mL of the supernatant was transferred to a 100-mL Erlenmeyer flask containing 25 mL of fresh ACE medium and incubated at 30°C with shaking at 160 rpm for 2 days.

### Shotgun metagenome sequencing

The shotgun metagenome sequencing of the enrichment cultures was described in the associated study^8^. Briefly, DNA was extracted from biomass pelleted from the enrichment cultures using the DNeasy UltraClean Microbial Kit (Qiagen, Hilden, Germany). Oxford Nanopore DNA libraries were prepared with the Native Barcoding kit 24 V14 (Oxford Nanopore Technologies, Oxford, UK) and sequenced on two R10.4.1 flow cells using the Oxford Nanopore PromethION 2 Solo platform. Basecalling using the SUPv4.3 super-accuracy model and demultiplexing of the Nanopore reads were performed with Dorado v0.5.3 (Oxford Nanopore). Additional processing including detection and splitting of concatenated read pairs, quality filtering of reads (mean quality score > Q15 and length > 1,000 bp), and removal of lambda DNA sequences was done using duplex-tools (https://github.com/nanoporetech/duplex-tools) and chopper^18^.

### MAG recovery, taxonomic classification and presence of antibiotic resistance genes

The filtered sequence reads were assembled on a per-sample basis using Flye v2.9.3^19^. All contigs were initially taxonomically classified with GTDB-Tk v2.1.0^20^ and CAT^21^ as described in the associated study^8^. Contigs classified as *Acinetobacter* were analyzed in the associated study^8^. The remaining non-*Acinetobacter* contigs were assessed for completeness and contamination using CheckM2^22^ and those with completeness > 50% and contamination < 10% (i.e. representing at least medium-quality MAGs,^10^) were retained for the present study. These MAGs were further dereplicated at the strain level, defined here as 99% average nucleotide identity, using dRep v3.4.0^23^ and their taxonomic status was re-checked using the GTDB-Tk v2.6.1^20^ against GTDB release R10-RS226^24^. The multiple-sequence alignment generated by GTDB-Tk was used to construct a genome-based phylogenetic tree with FastTree v2.2^25^. Branch lengths were transformed into an ultrametric tree using the penalized-likelihood method implemented in the chronos function of the ape package^26^ with λ = 1. The tree was visualized using the ggtree package^27^ in R v4.4.3^28^. Ribosomal regions were annotated with pybarrnap v0.5.0 (Seemann, T.; https://github.com/tseemann/barrnap) in accurate mode and at least 80% completion. Transfer RNAs were annotated with tRNAscan-SE v2.0.11^29^ with default values for bacterial genomes. The presence of antibiotic resistance genes was predicted using Abricate v1.2.0 (Seemann, T.; https://github.com/tseemann/abricate) with the NCBI AMRFinderPlus database^30^ (accessed 5 December 2025).

#### Data Records

The 84 single-contig, strain-level-dereplicated MAGs are available at the National Center for Biotechnology Information (NCBI), URL https://www.ncbi.nlm.nih.gov/, under the accession numbers JBXSAF000000000–JBXSDK000000000. Summary statistics for the MAGs, together with their individual accession numbers, taxonomy classification, and antibiotic resistance genes, are available in Supplementary Table 1.

#### Technical Validation

To prevent sample contamination, sterile tools and gloves were used when collecting each individual sample. Enrichment cultures were performed under sterile conditions, using a laminar flow hood. Extracted DNA and the individual and pooled libraries were quality-checked on an 12000 kit Agilent 2100 Bioanalyzer using the Agilent DNA 12000 Kit (Agilent Technologies, Santa Clara, CA, USA). Metagenomic reads underwent multiple quality control steps, including removing low-quality reads, adapters and lambda DNA with duplex-tools and chopper^18^. All MAGs presented here consist of a single contig, which largely reduces the risk of binning-derived chimerism, and were dereplicated at 99% average nucleotide identity using dRep^23^. Genome quality was assessed with CheckM2^22^, and only MAGs with completeness > 50% and contamination < 10% were retained, corresponding to at least medium-quality MAGs according to the standards proposed by Bowers et al.^10^.

## Supplementary Material

This is a list of supplementary files associated with this preprint. Click to download.

- SupplementaryTable1final.xlsx

## Data Availability

No custom scripts were used to generate the dataset. All analyses were performed using publicly available software, and the relevant tool versions and parameters are described in the Methods section.

## Data Availability

The 84 single-contig, strain-level-dereplicated MAG sequences are available at the National Center for Biotechnology Information (NCBI), URL https://www.ncbi.nlm.nih.gov/, under the accession numbers JBXSDF000000000, JBXSDE000000000, JBXSDD000000000, JBXSDC000000000, JBXSDB000000000, JBXSDA000000000, JBXSCZ000000000, JBXSCY000000000, JBXSCX000000000, JBXSCW000000000, JBXSCV000000000, JBXSCU000000000, JBXSCT000000000, JBXSCS000000000, JBXSCR000000000, JBXSCQ000000000, JBXSCP000000000, JBXSCO000000000, JBXSCN000000000, JBXSCM000000000, JBXSCL000000000, JBXSCK000000000, JBXSCJ000000000, JBXSCI000000000, JBXSCH000000000, JBXSCG000000000, JBXSCF000000000, JBXSCE000000000, JBXSCD000000000, JBXSCC000000000, JBXSCB000000000, JBXSCA000000000, JBXSBZ000000000, JBXSBY000000000, JBXSBX000000000, JBXSBW000000000, JBXSBV000000000, JBXSBU000000000, JBXSBT000000000, JBXSBS000000000, JBXSBR000000000, JBXSBQ000000000, JBXSBP000000000, JBXSBO000000000, JBXSBN000000000, JBXSBM000000000, JBXSBL000000000, JBXSBK000000000, JBXSBJ000000000, JBXSBI000000000, JBXSBH000000000, JBXSBG000000000, JBXSBF000000000, JBXSBE000000000, JBXSBD000000000, JBXSDK000000000, JBXSDJ000000000, JBXSDI000000000, JBXSDH000000000, JBXSDG000000000, JBXSBC000000000, JBXSBB000000000, JBXSBA000000000, JBXSAZ000000000, JBXSAY000000000, JBXSAX000000000, JBXSAW000000000, JBXSAV000000000, JBXSAU000000000, JBXSAT000000000, JBXSAS000000000, JBXSAR000000000, JBXSAQ000000000, JBXSAP000000000, JBXSAO000000000, JBXSAN000000000, JBXSAM000000000, JBXSAL000000000, JBXSAK000000000, JBXSAJ000000000, JBXSAI000000000, JBXSAH000000000, JBXSAG000000000, JBXSAF000000000. Summary statistics for the MAGs, together with their corresponding accession numbers, taxonomy classification, and antibiotic resistance genes, are available in Supplementary Table 1.
